# Seasonal variations, source apportionment, and risk assessment of polycyclic aromatic hydrocarbons (PAHs) in sediments from Klip River, Johannesburg, South Africa

**DOI:** 10.1007/s10661-025-13724-0

**Published:** 2025-02-08

**Authors:** Samuel Makobe, Mathapelo P. Seopela, Abayneh A. Ambushe

**Affiliations:** Department of Chemical Sciences, University of Johannesburg, P.O. Box 524, Johannesburg, Auckland Park 2006 South Africa

**Keywords:** PAH risk assessment, River sediments, Source identification, Spatiotemporal variations, Zebrafish embryo development test

## Abstract

The widespread occurrence of polycyclic aromatic hydrocarbons (PAHs) in water bodies is a significant cause of concern due to their persistence and toxicity. This study aimed at assessing the qualitative and quantitative profile of 16 EPA PAHs present in sediment collected from nine sites along the Klip River, Johannesburg, South Africa. The target compounds were extracted from sediment by microwave-assisted extraction (MAE). The extraction method accuracy was evaluated by analyzing certified reference material of sediment (CRM-104). The total concentration of PAHs detected in sediment samples ranged from 1.46 to 7.41 mg/kg. High molecular weight (HMW) PAHs were found to be in higher concentrations for both low flow (24.5 mg/kg) and high flow (19.4 mg/kg) seasons compared to the lower molecular weight (LMW) PAHs, which had 11.1 mg/kg for low flow and 5.63 mg/kg for high flow seasons. For identification of source of PAHs in sediments, the diagnostic ratio was employed, which revealed the PAHs were from petrogenic and pyrogenic sources. Toxicity test using zebrafish embryo development test (ZFET) resulted in high mortality, low hatch rate, and high malformations, which indicated the contamination of sediments from the Klip River. These findings suggest that exposure to this water system poses risks to the environment, aquatic life, and humans who depend on it.

**Supplementary Information:**

The online version contains supplementary material available at 10.1007/s10661-025-13724-0.

## Introduction

Water resources around the world are increasingly in danger due to the contamination of water bodies by the discharge of wastewaters from domestic and industrial sources, mines, and other causes (Gqomfa et al., 2022; Sibiya, 2012). In South Africa, the function of ecosystem, public health, and water quality has been distressed due to pollution (Edokpayi et al., 2017; Mogashane et al., 2020). Water supplies are under increasing pressure due to pollution and resource depletion, due to rising demand because of the increase in population, urbanization, and industrialization. Polycyclic aromatic hydrocarbons (PAHs) are defined as a group of ubiquitous organic pollutants composed of fused aromatic rings made up of carbon and hydrogen atoms (Olisah et al., 2021). At room temperature, PAHs are solids with low volatility and relatively high molecular weights and are soluble in a range of organic solvents but insoluble in water (Chetty et al., 2021; Pheiffer et al., 2018). The position and number of rings influence chemical and physical properties, lipophilicity, interactions with humans and other organisms living in various environments, and environmental behavior (Duran & Cravo-Laureau, 2016; Zoveidadianpour et al., 2023).

Upon entering the aquatic environment, PAHs tend to remain in the water and later adsorb to the sediments. The exchange of PAHs between water and sediment plays a vital role in determining their environmental fate as well as their potential impact on aquatic organisms (Jesus et al., 2022). PAHs bio-accumulate in food chains and can be transferred to different areas of the globe due to their semi-volatility character. Even though PAHs are not accurately poisonous to biota and animals, their buildup across food chains puts predators in danger of toxicity because they are hard to excrete from the body. High-concentration exposure is extremely unusual in people, but long-term exposure to low concentrations can cause chronic sickness in humans, including immunological disorders, allergies, reproductive alterations, hypersensitivity, cancer, and neurotoxicity (Klingberg et al., 2022). Hence, PAHs’ assessment to ensure a protected environment for the health of humans and sustainability is essential as a means of reducing their production (Patel et al., 2020). The World Health Organization (WHO) has identified and classified PAHs as a priority group of environmental pollutants due to their possible and persistent harmful effects on aquatic animals and humans. The primary sources of PAHs include the pyrogenic and petrogenic processes. This is a consequence of the higher possibility of PAH production from both industrial and heavy usage of fossil fuels in urban environments, including the health risks associated with them. Therefore, it is critical to examine and track the levels of PAHs as a form of monitoring them regularly (Howard et al., 2021; USEPA, 2008).

The Klip River is one of the most economically important wetlands in South Africa, which is located in the southern part of Johannesburg. It serves as a local supply of water and evolved as a purifier of pollutants from the Witwatersrand Basin’s diverse anthropogenic activities (Awe et al., 2020). The Klip River wetland ecosystem could be contaminated with PAHs stemming from a variety of sources, including acid mine runoff, industrial and municipal waste, untreated and treated sewage, automobile emissions, the burning of garbage, and the burning of coal in industrial complexes near the river (Chetty et al., 2021). Additional PAH investigation into the Soweto and Lenasia regions was motivated by the discoveries made in the research conducted by Olasupo and Buah-Kwoe (2021) and Pheiffer et al. (2018), which revealed high levels of PAHs in water and sediments in those areas.

The Organization for Economic Co-operation and Development (OECD) has released acute chemical toxicity test guidelines and recommends using zebrafish in ecotoxicity testing methods for aquatic environments since they fulfill the OECD guidelines (Zhao et al., 2021). Zebrafish testing has become the gold standard for determining the toxicity of water, effluents, and sediments (Hussain et al., 2018). Several biomarker responses in zebrafish have been used as a model system due to their effectiveness for evaluating conventional and emerging contaminants’ ecotoxicological effects globally (Ebele et al., 2017; Hirondart et al., 2020). When using fish as bio-monitors in water and sediment quality assessments, many benefits have been seen, as they can detect changes in the water bodies and respond to harmful compounds likewise higher invertebrates and mammals (Ngubo et al., 2021). Hubrecht and Carter (2019) suggested an alternative, creative method of refining, reducing, and replacing animal tests using in vitro methods. According to Hedgpeth et al. (2019), the fish embryo toxicity test (FET) is a viable substitute for the use of acute fish toxicity. Studies have shown that embryos of zebrafish can be used to anticipate toxicity and developmental changes in humans and animals upon exposure to toxic chemicals (Schiwy et al., 2014; Seopela et al., 2016; Tartaglione et al., 2023; Wang et al., 2021).

There are limited studies that focused on the investigation of PAHs in sediment along the Klip River, their toxicity, and the harmful effect they pose on aquatic animals and humans (Moja et al., 2013; Olasupo & Buah-Kwoe, 2021; Pheiffer et al., 2018; Rimayi et al., 2017). This study aims to assess the spatiotemporal variations of the 16 United States Environmental Protection Agency (US EPA) PAHs, which have been priority pollutants, in sediment from the Klip River Catchment, which passes through Lenasia and Soweto. This area is known to be a heavily populated urban area, where elevated concentrations of PAHs have already been reported in literature (Pheiffer et al., 2018). Furthermore, this study aims to conduct a toxicity assessment of the collected river sediments, using the zebrafish embryo development test (ZFET), which had not been conducted in previous studies in the study area.

## Experimental

### Study area description

**Fig. 1:**
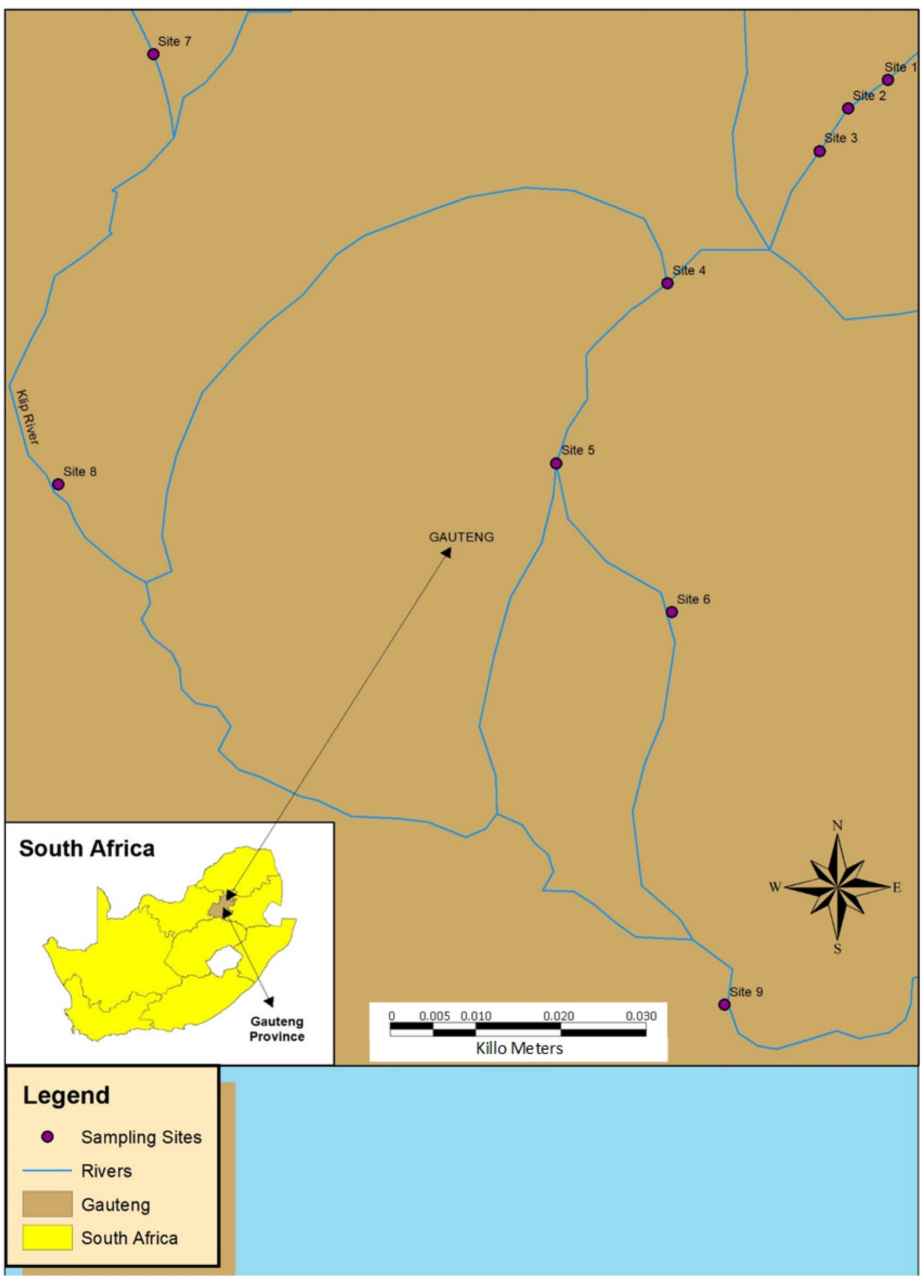
A map of the nine selected sampling sites (sites 1 to 9) along the Klip River

Klip River, situated in the south of the Witwatersrand region of Johannesburg, South Africa, along with its tributaries, has been regarded as one of the most extensively contaminated rivers in the country (Fig. 1) (Pheiffer et al., 2014). It is the sub-catchment of the upper Vaal River Water Management Area (WMA) (Pheiffer et al., 2014). This study area was selected due to the widespread exposure to untreated wastewater discharge from informal settlements and municipal, industrial, and mining operations in the surrounding area (Olisah et al., 2021). In addition, leaking sewage systems and irresponsible littering from settlements in rural areas along the Klip River and its tributaries also contribute to domestic discharge. Point sources of pollution in the catchment primarily originate from mining activities and wastewater treatment plants (WWTPs), while diffuse pollution is mainly associated with informal settlements and the presence of outdated mine slime dams or waste dumps. The water utility, Rand Water, distributes clean water sourced from the river to numerous municipalities situated within the catchment area (Chetty et al., 2021). Primary consumers of water include manufacturing industries (Chamdor Industrial area) engaged in activities like product packaging and the production of roofing and cladding materials, as well as three WWTPs (Goudkoppies, Olifantsvlei, and Bushkoppies) and mining operations (Central Gold Recovery and Ergo) (Pheiffer et al., 2014). The upper catchment of the Klip River is the most significant area for mining activities, including gold, base metals, and industrial minerals. Water from the catchment is also used for agricultural purposes such as watering livestock and irrigating crops (Chetty et al., 2021).

### Sampling, sample preservation, and storage

Sediment samples were collected from nine selected sampling sites, sites 1 to 9, along the Klip River (Fig. 1) in October 2021 and July 2022, representing high and low flow seasons, respectively. For the identification of seasons, sample types, and sampling sites along the Klip River, a system of abbreviations was employed. The first letter denotes the season of sample collection, the second letter indicates the sample type, and the number signifies the specific site along the river where the sample was collected. For example, SS1 denotes a sediment sample collected during the low flow season from site 1, while WS3 represents a sediment sample collected at site 3 during the high flow season. Sediment samples were collected using a 1.5-m auger corer with a 20 × 10 cm cylinder and transferred into a new, clean glass jar (375 mL). Sediment samples were dried and ground with an agate mortar and pestle to a fine powder, then sieved through a 250-µm sieve, and stored in resealable plastic ziplock bags until extraction. All samples were collected in triplicate.

### Reagents and consumables

High-purity solvents were used to prepare all the solutions. The purity of reagents and their suppliers are presented in detail in the Supplementary Material.

### Apparatus and instrumentation

The details of the apparatus and instrumentation used in this study are presented in the Supplementary Material.

### Extraction of PAHs from river sediments

The MAE method applied was adopted by Seopela et al. (2016), with modifications. The MAE was done using a CEM MARS microwave extraction system. About 5 g of a freeze-dried, ground, and sieved (< 250 µm) sediment sample was weighed and transferred to a 110-mL Teflon extraction vessel. Thereafter, 30 mL of (1:1) hexane and acetone was added. The vessels were sealed, loaded onto the turntable, and placed in the microwave extraction system. The extraction of PAHs from river sediments was realized by setting the power at 1600 W, with a 15-min temperature ramp to 115 °C, held for 30 min and a total run time of 57 min. Once the extraction vessels had cooled, the extracts were transferred into amber vials. About 0.3 g of activated copper, prepared by combining the copper powder with 2 mL of nitric acid, was added to each extract. The extracts were subsequently washed with de-ionized water and acetone. Thereafter, the extracts were transferred to a 250-mL round-bottom flask. A rotary evaporator was used to concentrate the extracts to approximately 3 mL. The 3-mL extract was then passed through a 5-mL syringe filter packed with anhydrous sodium sulfate and stored in a 4-mL vail. Under a gentle stream of nitrogen, each extract was then evaporated close to dryness. Then, 10 µL of 1 mg/L of a mixed internal standard solution containing Per-D12, Chr-D12, Ace-D10, and Nap-D8 was added to each extract and made up to 1000 µL with dichloromethane. The resulting solution was mixed using a Vortex-Genie and transferred to a 1.5-mL vial using an automatic pipette prior to GC-FID analysis. A CRM-104 containing PAHs was used to confirm the accuracy of the method. The percentage recovery was calculated for each PAH to confirm the accuracy of the method.

### Sample analysis by GC-FID

The identification and determination of the target compounds were conducted by GC-FID. Samples (2 µL) were introduced using a splitless injection mode at a temperature of 280 °C. A temperature program initially at 50 °C, held for 3 min, was raised at 5 °C per minute to 75 °C, maintained for 3 min. Furthermore, the program was raised at 10 °C per minute to 200 °C, maintained for 5 min. Lastly, it was raised at 5 °C per minute to 270 °C, maintained for 6 min. The detailed operating conditions are outlined in Table A1 of the Supplementary Material.

**Table 1:** The diagnostic PAH ratio of sediment samples collected from the Klip River during the high and low flow seasons

	Phe/Ant	Ant/(Phe + Ant)	Flu/Pyr	Flu/(Flu + Pyr)	BAnt/(BAnt + Chr)
SS1	0.195	0.837	0.985	0.496	0
SS2	0.155	0.866	2.00	0.667	0
SS3	0.223	0.818	0.681	0.405	0.0897
SS4	0.121	0.892	0.793	0.442	0
SS5	0.106	0.904	0.530	0.347	1.00
SS6	0.090	0.917	0.862	0.463	0
SS7	0.140	0.877	0.441	0.306	0.329
SS8	0.081	0.925	1.37	0.578	0.255
SS9	0.0441	0.958	0.383	0.277	0.178
WS1	0.750	0.571	0.064	0.0600	0
WS2	0	0	0.239	0.193	0
WS3	0	1.00	0	1.00	0
WS4	0.117	0.895	0.235	0.190	0
WS5	0.306	0.766	0.305	0.234	0.249
WS6	0.083	0.924	0.130	0.115	0.395
WS7	0.383	0.723	0.229	0.187	0.164
WS8	0.227	0.815	1.57	0.610	0.161
WS9	0.196	0.836	0.113	0.101	0.260

### Analytical figures of merit

The limit of detection (LOD) and limit of quantification (LOQ) were calculated as three and ten times the standard deviation of the average of all prepared reagent blank solutions, respectively. To monitor the precision of all sets of the results, the standard deviation (SD) and percentage relative standard deviation (% RSD) were calculated. The linearity of calibration curves and the accuracy of the method were evaluated. The details of method validation are presented in the Supplementary Material along with the tables A2 and A3.

### Determination of PAHs in river sediments by GC-FID

The PAHs in sediments were identified (Fig. A1) and quantified by GC-FID. The details are presented in the supplementary material.

### Statistical analysis

One-way analysis of variance (ANOVA) was employed to evaluate the statistical significance of variations of the mean concentrations of PAHs in river sediments from the Klip River. The data evaluation was conducted using SPSS Statistics software, with a significance level of *p* = 0.05 being used to assess statistical significance. The variation was considered statistically significant if *p* < 0.05 and insignificant if *p* > 0.05 at a 95% confidence level.

### Zebrafish embryo development test

The test procedure in this study was conducted in line with the OECD 236 FET test (OECD, 2013). Adult zebrafish were fed with 20 mg of zebrafish food (ZM-400) granules using a Trotone automatic feeder. Male and female long-finned adult zebrafish were housed in a multi-linking ZebTec housing system with a 12:12-h light–dark cycle and kept at a constant temperature of 26 ± 1 °C. Breeding was carried out using the standardized operating methods developed by the National Aquatic Bioassay Facility at Northwest University, Potchefstroom campus, South Africa. The day before mass spawning, fish were not fed, and males and females (1:2) were separated into different tanks (14 males and 28 females). Thirty-six adult fish were inserted in an iSPAWN Zebrafish tank at a temperature of 27.9 °C, where females were kept at the bottom and males were confined to the top of the tank by a separating net. Mating behavior was induced by elevating the breeding basket, removing the divider and air stones, and gradually adjusting the water levels. For acclimatization, the fish were subsequently lowered halfway down in 10-min stages, removed, and afterwards put back into the housing tanks. The fertilized embryos were retrieved using the bottom valve of the iSPAWN breeding tank. A stereomicroscope was used to separate viable eggs from non-viable eggs. Identical stage fertilized eggs (2.5 h post-fertilization (hpf)) were then selected for the embryo test in an E3 medium (reconstituted water) made from 0.17 mM KCl, 0.33 mM CaCl_2_·2H_2_O, 5.0 mM NaCl, and 0.33 mM MgSO_4_·7H_2_O prepared in accordance with ISO 7346 at a pH of 7.4 and conductivity of 592 µS.

The sediment contact assay used in this study was adopted from the study by Seopela et al. (2016). Embryo medium was used as a negative control, while 4.0 mg/L of 3.4-dichloroaniline (3.4-DCA) was used as a positive control. About 2.00 g of each sediment sample was placed in each well of a 12-well microtiter plate. This was followed by the addition of 3.00 mL of the E3-medium. To ensure that the samples on the plates were not tempered before the embryos were inserted, they were then placed in an incubator set at a temperature of 26 ± 1 °C. A total of *n* = 36 embryos (3 per well) were exposed to the sediment samples. An oxygen-permeable self-adhesive film was used to seal the plates to avoid evaporation of the test solutions. The developmental characteristics of the embryos were monitored for 96 hpf at 24 hpf intervals. A non-intrusive video method adopted from the study conducted by Kataba et al. (2022) was employed to examine embryos or larvae that had hatched. The embryo activity was evaluated at 24 hpf by recording videos for 1 min with a stereomicroscope and a remote-controlled camera. The average frequency of movement (burst activity) and the number of movements per minute (burst count/min), recorded for 1 min at 24 hpf, were used as indicators of embryo activity. The heart rate and blood flow were monitored at 72 hpf. The blood flow was monitored by focusing on the caudal hemopoietic region close to the cloaca opening for 30 s. All videos were analyzed with DanioScope V1 software (Noldus Information Technology, Wageningen, Netherlands).

## Results and discussion

### Levels of PAHs in the river sediments during the low and high flow seasons

**Fig. 2:**
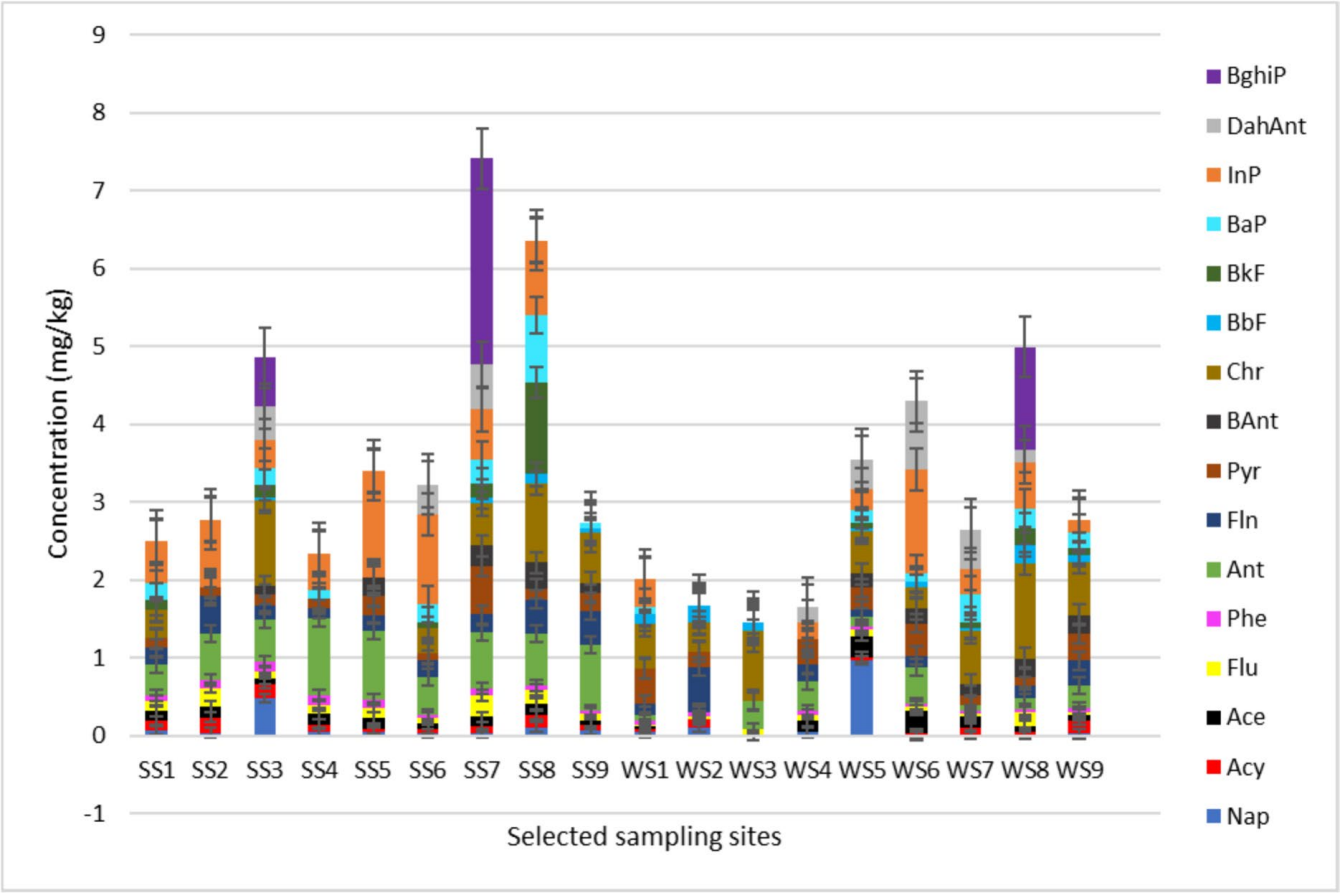
The distribution of PAHs in sediment samples from the Klip River during the high and low flow seasons

The total concentrations of PAHs in river sediments collected during the low and high flow seasons ranged from 2.35 to 7.41 mg/kg and 1.46 to 4.99 mg/kg, respectively (Fig. 2). The highest PAH concentration values during the low and high flow seasons were observed at sites 7 and 8 and the lowest at sites 3 and 4 (Fig. 2). The elevated PAH levels at sites 7 and 8 were primarily attributed to presence of industrial activities, sewage leaks, agricultural runoff, and resident littering near the study area. However, at sites 3 and 4, no industrial or agricultural activities were noted near the study area. The only sources of pollution appeared to be littering by residents and sewage leaks from a nearby informal settlement. The concentration of BAnt at sites 1 to 6 was lower than the LOD (0.0454 mg/kg), including the following PAHs at the indicated sites within the brackets: Chr (2, 4, and 5), BbF (1, 2, 4, 5, and 6), BkF (2, 4, and 5), BaP (2 and 5), InP (9), DahAnt (1, 2, 4, 5, 8, and 9), and BghiP (1, 2, 4, 5, 6, 8, and 9). However, the concentrations of PAHs in sediment samples during the high flow season were above LOD in most of the sites except Nap at sites 3, 6, and 9, Acy (sites 3, 4, 6, and 9), Ace (sites 1, 2, 3, and 4), Phe (sites 3 and 7), Ant (site 2), Fln (sites 3 and 7), Pyr (site 3), BAnt (site 4), Chr (site 4), BbF (site 4), BkF (sites 1, 2, 3, 4, and 6), BaP (sites 2, 3, and 4), InP (sites 2 and 3), DahAnt (sites 1, 2, 3, and 9), and BghiP (sites 1, 2, 3, 4, 5, 6, 7, and 9).

The LMW PAHs ranged from 0.741 to 6.13 mg/kg and from 0.340 to 1.90 mg/kg for the low and high flow seasons, respectively. The lowest individual LMW PAH during the low and high flow seasons was observed at sites 7 and 8 (Nap), and the highest was at sites 4 and 6 (Ant). However, the HMW PAHs ranged from 0.279 to 6.37 mg/kg and from 0.424 to 5.25 mg/kg during the low and high flow seasons. The individual HMW PAH present at the lowest was observed at sites 2 and 7 (BbF), and the highest was at sites 5 and 6 (InP). The combined total concentrations of HMW PAHs across all the sites during the low and high flow seasons were higher compared to the LMW PAHs. This is attributed to HMW compounds being more lipophilic than LMW compounds, leading to higher concentrations in river sediments compared to LMW PAHs (Pheiffer et al., 2018). During the low flow season, sites 3, 7, and 8 exhibited the highest levels of pollution, while during the high flow season, sites 5, 6, and 8 showed significant pollution. The irregulated discharge of wastewater, diverse sewage discharges, and the numerous activities of various industries in the river’s vicinity are only a few of the aspects that make it challenging to comprehend the temporal variation of PAHs in the river (Meland et al., 2019). However, the spatial variation observed in the concentration of detected PAHs in river sediments per site is clear because their concentration increases from upstream to downstream, which is the same conclusion drawn by Zhu et al. (2023). The study done by Pheiffer et al. (2018) in the same study area (2017) reported a total PAH concentration ranging from 0.274 to 5.37 mg/kg, which shows an increase in the level of PAHs detected from the Klip River in this study. The observed increase in the concentration of PAHs can be attributed to an increase in activities contributing towards water pollution, such as municipal, industrial, and agricultural waste; sewage discharges; and the high traffic taking place around the study area as the population and urbanization have been increasing with time.

An ANOVA was performed on the detected PAH concentrations in sediment samples at a 95% confidence level for the high and low flow seasons. The results showed a significant difference at a 95% confidence level between the levels of PAHs of the river sediments for the high and low flow seasons (*p*-value = 0.036). This implies that the detected concentrations of PAHs in river sediments varied between the two seasons. As a result, based on the statistical analysis, one can conclude that there was a substantial difference between the PAH concentrations detected in river sediments during the high and low flow seasons. The observed seasonal fluctuation likely stems from diverse factors, such as differences in the origins of PAHs at each site, variations in biological processes, variations in atmospheric deposition, dynamics of hydrological movement, and the mitigating effect of dilution effects (Mukhopadhyay et al., 2023; Zheng et al., 2020).

### Identification of sources of PAHs in sediment samples

**Fig. 3:**
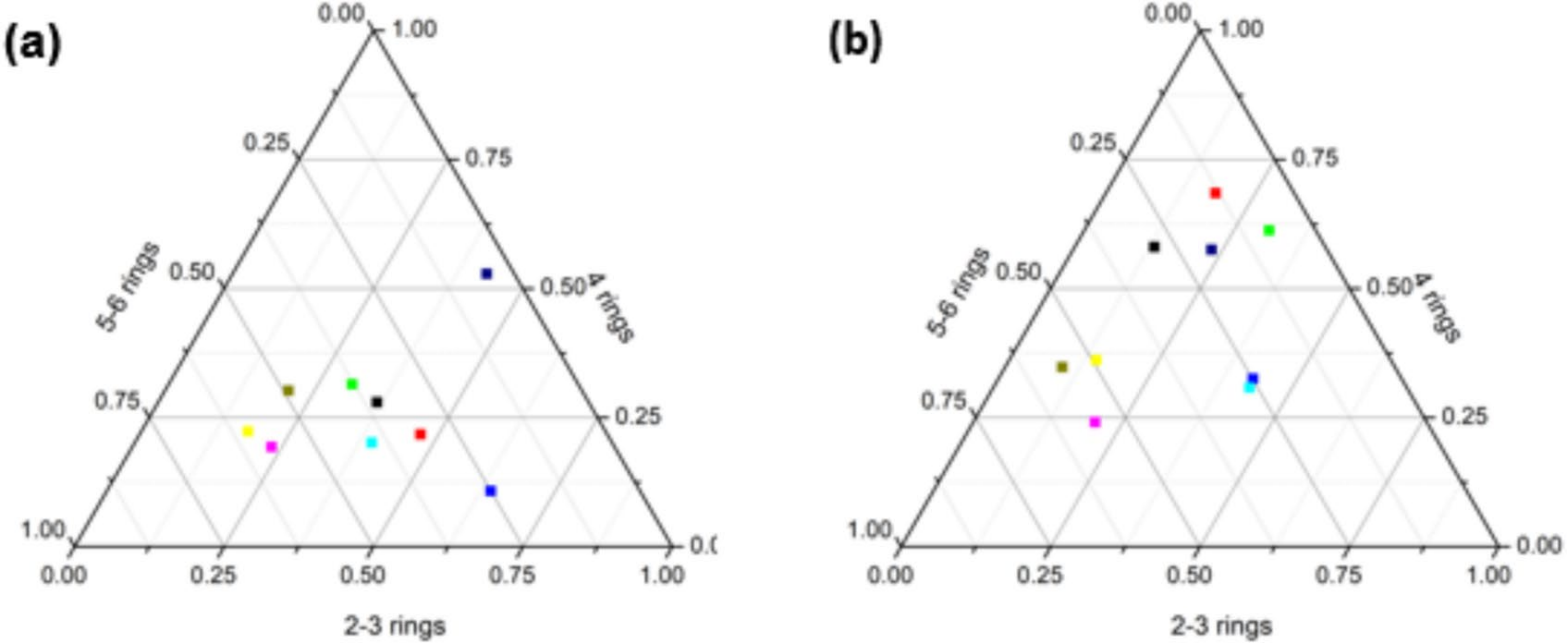
Ternary plots with percentage composition of detected PAHs in river sediments during the low (**a**) and high flow (**b**) seasons at the Klip River

The sources of PAHs were first deduced by grouping the compounds according to the number of rings, where low, middle, and higher molecular weight PAHs were represented by 2 to 3 rings, 4 rings, and 5 to 6 rings (Aralu et al., 2023). The most abundant and dominant concentrations during the low and high flow seasons were observed in the low and higher molecular PAHs, as well as in the middle and higher molecular PAHs. During the low flow season, the sum of 2 to 3 ring PAHs contributed 31.23% of the total 16 PAHs; 4 ring PAHs contributed 26.44%; and 5 to 6 ring PAHs contributed 42.33% (Fig. 3a). However, during the high flow season, the following contributions were observed: 2 to 3 ring PAHs (22.50%), 4 ring PAHs (40.55%), and 5 to 6 ring PAHs (36.96%) (Fig. 3b). The obtained results correlate well to those detected by GC-FID, as sediment samples collected during the low flow season were found to be more toxic compared to those collected during the high flow season. The composition of the 16 PAHs detected in both seasons is shown in Fig. 3a and b.

It is feasible to establish whether the PAHs originate from petrogenic or pyrogenic sources by looking at the ratio of HMW to LMW PAHs. To determine potential origins of PAHs in sediment samples for high and low flow seasons, the diagnostic ratios Flu/(Flu + Pyr), Ant/(Ant + Phe), and BAnt/(BAnt + Chr) were employed, respectively (Table 1) (Katsoyiannis et al., 2007; Ravindra et al., 2008; Tobiszewski & Namieśnik, 2012; Zhang et al., 2008). To precisely investigate petrogenic or pyrogenic sources of PAHs in sediments, the ratio Ant/(Ant + Phe) was calculated. A ratio larger than 0.1 indicates a pyrogenic source, while a ratio less than 0.1 indicates petroleum contamination (Katsoyiannis et al., 2007). Based on the study by Zhang et al. (2008), Flu/Pyr and Flu/(Flu + Pyr) ratios are further markers for determining the source of PAH pollution in sediments. Additionally, the pyrogenic source from the combustion of grass, wood, or coal is proposed if Flu/(Flu + Pyr) > 0.5. PAHs are primarily produced when petroleum is burned if the ratio of Flu/(Flu + Pyr) is between 0.4 and 0.5. If the ratio is below 0.4, petroleum contamination is hypothesized. Additionally, a ratio of BAnt/(BAnt + Chr) of less than 0.2 typically denotes a petrogenic origin, 0.2 to 0.35 denotes a mixed petrogenic and pyrogenic origin, and > 0.35 denotes a pyrogenic origin.

During the low flow season, the Ant/(Ant + Phe) ratios ranged from 0.818 to 0.958 with a mean value of 0.888, indicating that the detected PAHs were from pyrogenic sources, while the Flu/(Flu + Pyr) ratios ranged from 0.277 to 0.667 with a mean value of 0.443, indicating that the PAHs were mostly from the combustion of petroleum. The BAnt/(BAnt + Chr) ratios ranged from 0 to 1.00, with a mean value of 0.206, indicating a mixed petrogenic and pyrogenic origin. However, during the high flow season, the Ant/(Ant + Phe) ratios ranged from 0 to 1.00 with a mean value of 0.726, which also indicated that the detected PAHs were from pyrogenic sources, while the Flu/(Flu + Pyr) ratios ranged from 0.0600 to 1.00 with a mean value of 0.298, indicating petroleum contamination. The BAnt/(BAnt + Chr) ratios ranged from 0 to 0.395 with a mean value of 0.137, indicating petrogenic sources. Hence, at site 7 during the low flow season, activities taking place around the area that cause elevated levels of PAHs detected in sediment samples compared to other sites include industrial activities, sewage leaks, agricultural runoff, abandoned mine waste sites (closed solid waste site at Dobsonville), littering by residents, and excessive traffic flow near the site. The increase in PAH concentration detected at site 8 during the high flow season compared to the other sites might be due to the increase in activities leading to the production of PAHs, such as the Chamdor industrial area near the site, high traffic taking place on the bridge, and leaking sewage systems in the informal settlements around the selected site. In conclusion, the PAHs in the sediment samples collected from the Klip River during the high and low flow seasons originated from pyrogenic sources, petroleum contamination, and petrogenic sources.

### Zebrafish embryo test

The zebrafish embryos were exposed to the sieved sediment samples for 96 hpf. Major endpoints included %mortality rate, hatch rate, and malformations.

#### The effect of sediment samples on the hatch rate of zebrafish embryos

**Fig. 4:**
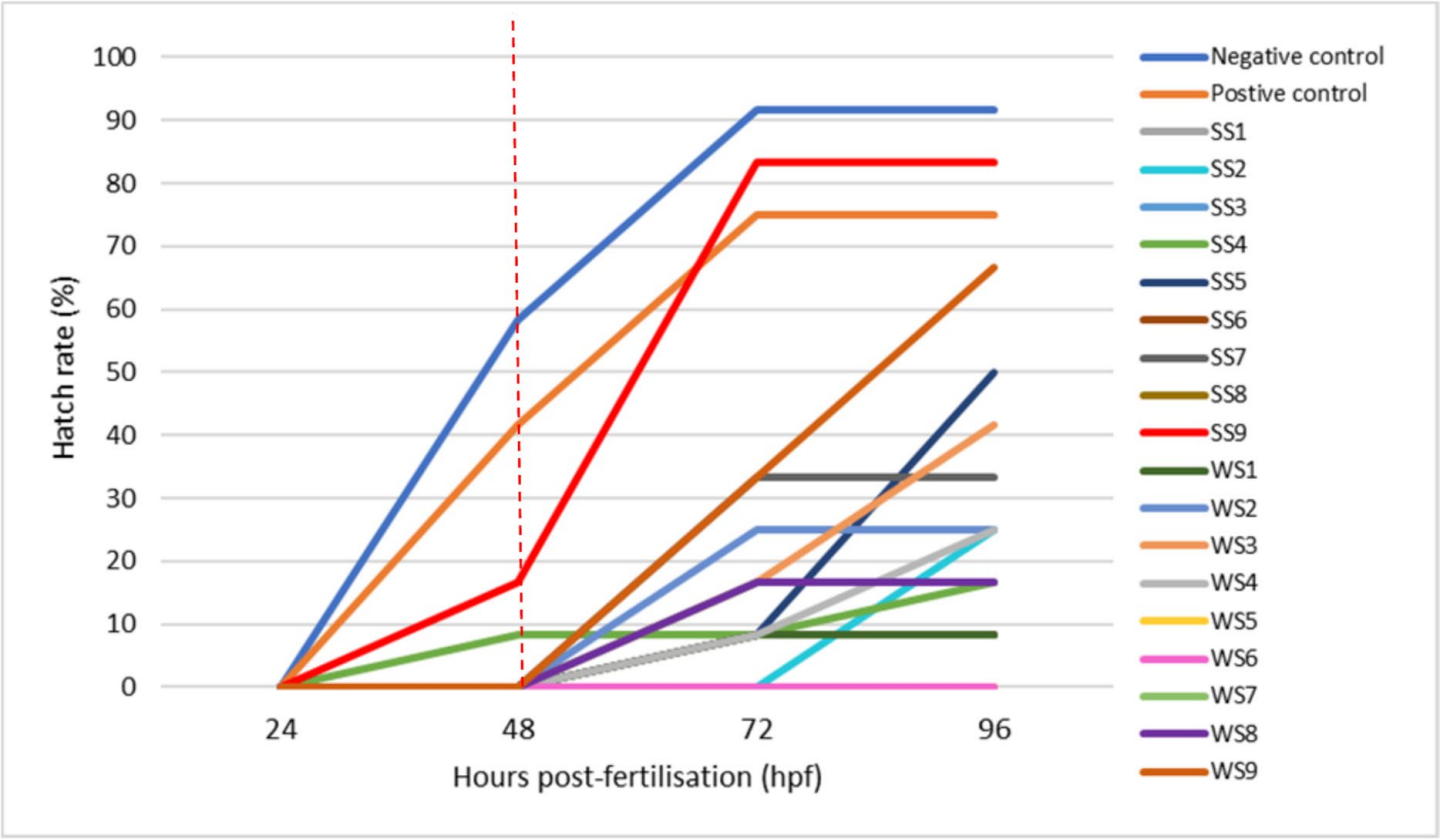
The hatch rates of zebrafish embryos exposed to sediments collected from sites 1 to 9 from the Klip River monitored for 96 hpf

The validity of the test was confirmed by exposing zebrafish embryos to a negative (E3 medium) and positive control (4.0 mg/L of 3.4-DCA). Embryos exposed to the negative control had high hatch rates during the test period (58.33% at 48 hpf and 91.67% at 72 to 96 hpf) compared to the positive control (medium with toxins), which showed a reduced hatch rate of 41.76% at 48 hpf and 75% at 72 to 96 hpf. According to the study conducted by Wan-Mohtar et al. (2021), the hatch rate of the zebrafish embryo begins at 48 hpf. On average, the hatching rate of embryos in all 9 sites at 24 to 96 hpf ranged from 0 to 45.83% (Fig. 4), with the least and highest values obtained for embryos exposed to SS1, SS8, and WS5 as well as WS6 and SS9. Only embryos exposed to SS9 hatched on the second day (˃10% significant) and had reached 83.33% by the end of the test period. Embryos exposed to SS1, SS8, WS5, and WS6 did not hatch for the whole test period (96 hpf), which showed a high level of pollution present at those sites, resulting in the observed lack of hatching. A study conducted by Bozinovic et al. (2021) indicated that zebrafish embryo exposure to PAHs induced developmental delays and reduced heart rates. Hence, the results illustrate that sediment samples collected from the above-mentioned sites can be regarded as class V, which indicates a serious environmental concern as hatching was not evidenced at those sites showing a high level of pollution that negatively affects the development of aquatic animals. The same conclusion made regarding the reduced hatch rate associated with the presence of detected PAHs in high concentrations was also reported by Viganò et al. (2020).

At 72 hpf, only 8.33% of the embryos hatched at SS3 to SS6, WS1, and WS4, 16.67% (WS3 and WS7 to WS8), 25% (SS2), and 33.33% (SS7 and WS9), which highlights the fact that highly polluted sediment samples negatively affect the hatch rate of the zebrafish embryos. Lastly, embryos exposed to SS3, SS6 to SS7, SS9, WS1 to WS2, and WS7 to WS8 at 96 hpf did not change in terms of their hatch rate, and a slight increase in hatch rate was observed in SS4 (8.33 to 16.67%), WS3 (16.67 to 14.67%), WS4 (8.33 to 25%), and WS9 (33.33 to 66.66%). The sediment samples from the Klip River are possible toxic PAH reservoirs, as embryos exposed to the sites with elevated PAH concentrations experienced reduced hatch rates. Since the hatch rate of zebrafish embryos is considered a sign of toxicity in environmental studies, sediment samples from the Klip River are considered to be contaminated and pose a serious environmental concern that needs to be monitored and reduced for the sustainability of the environment (Marara & Palamuleni, 2019). The hatch rate of zebrafish embryos exposed to sediment samples collected during the high and low flow seasons at each site during the exposure time interval is shown in Fig. 4.

#### Mortality rate of zebrafish embryos exposed to river sediments

**Fig. 5:**
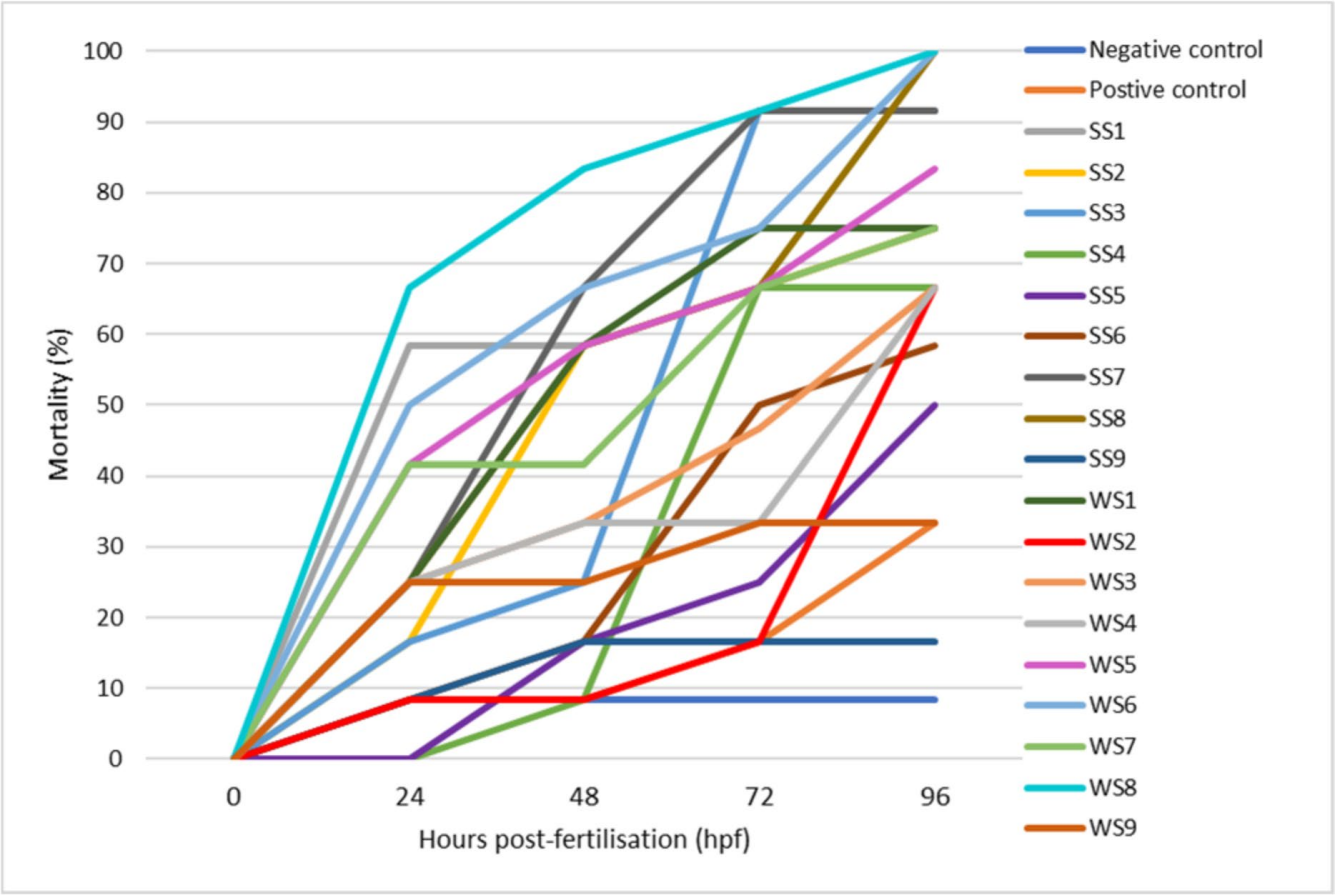
The mortality rate of zebrafish embryos exposed to the sediment collected from sites 1 to 9 from the Klip River monitored for 96 hpf

The validity of the test was verified by subjecting zebrafish embryos to both a negative control (E3 medium) and a positive control (4.0 mg/L of 3,4-DCA). Embryos exposed to the negative control showed a low average mortality rate of 8.33% throughout the test period, while a high average mortality rate of 33.33% was observed in embryos exposed to the positive control (medium with toxins). On average, the mortality rate of embryos in all 9 sites at 24 to 96 hpf ranged from 14.59 to 85.42% (Fig. 5), with the least and highest values obtained for embryos exposed to SS9 and WS8, respectively. To evaluate the toxicity of river sediments based on the mortality of the test organism, the toxicity criteria proposed by Persoone et al. (2003) using the percentage effect (PE) and the biotest were employed (Table A4; Supplementary Material).

The fish embryotoxicity test has already been used in earlier studies to determine the embryotoxic effects in native sediments (Adom et al., 2022; Marara & Palamuleni, 2019). It is evident that the mortality rate of zebrafish embryos exposed to all river sediments tends to rise as hpf increases during the test period (Fig. 5), and similar results were reported by Schiwy et al. (2014). Nonetheless, it is worth noting that the degree of mortality increase differs among the various sediment samples, indicating varying levels of toxicity.

**Fig. 6:**
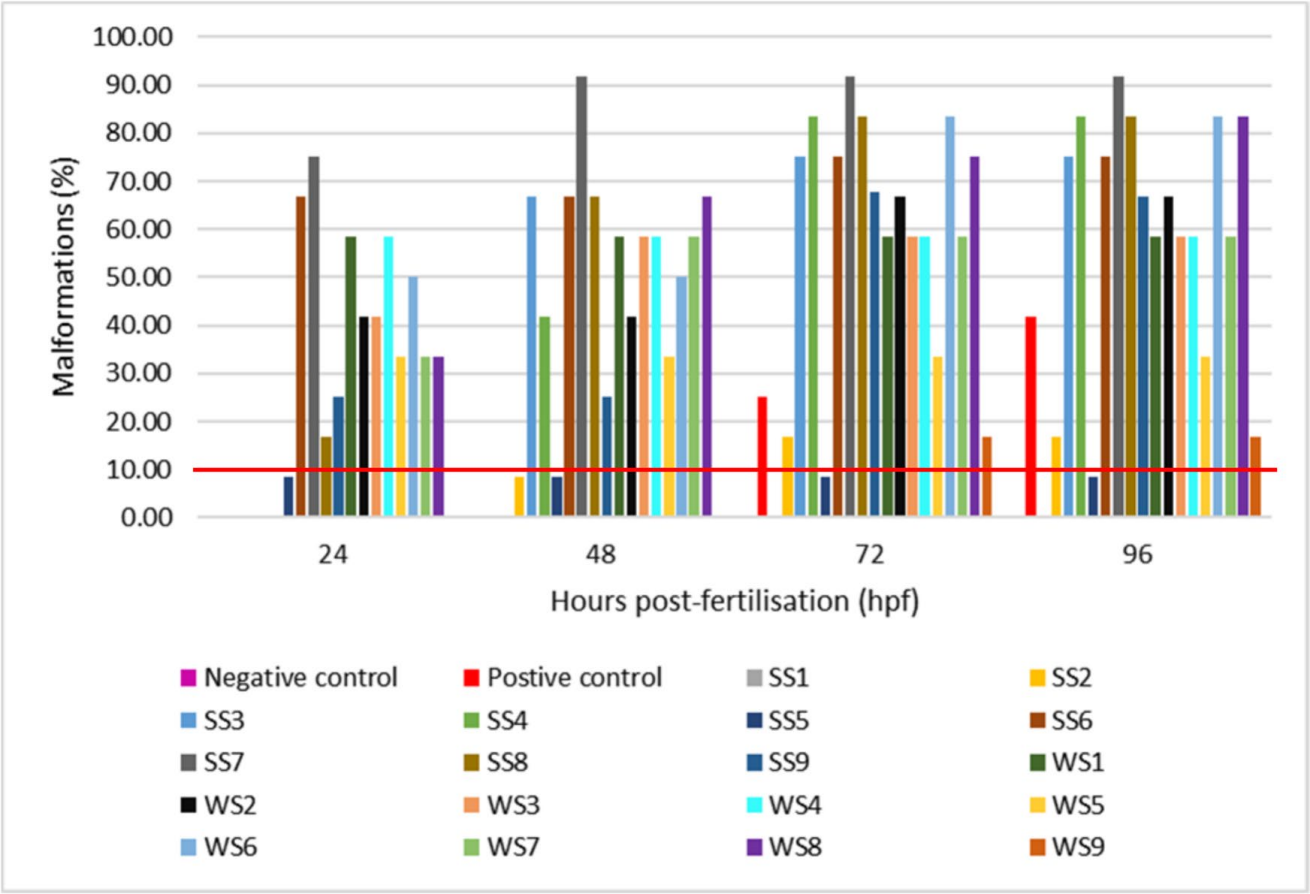
The percentage malformations of zebrafish embryos exposed to sediments collected from sites 1 to 9 from Klip River monitored for 96 hpf and significant malformations (over 10%) indicated by a horizontal red line

For the low flow season sediment samples, average mortality ranged from 22.92 to 68.75%. The highest mortality of zebrafish embryos was observed at SS7 (68.75%), SS1 (64.58%), and SS8 (62.50%). Sites SS1, SS7, and SS8 have been regarded as some of the sites highly contaminated with PAHs. The lowest mortality was observed at SS9 (14.59%) and SS5 (22.92%) (Fig. 6). Due to the observed high average mortality of embryos exposed to SS1 (64.58%), SS7 to SS8 (68.75 to 62.50%) (Fig. 5), one can conclude that sediment samples with elevated levels of PAHs induce high mortality (Adom et al., 2022; Marara & Palamuleni, 2019). The same results were reported by Seopela et al. (2016), after performing an intact sediment exposure on zebrafish embryos, for which high mortality was observed when embryos were exposed to low flow season sediment samples with high levels of PAHs. In addition, embryos exposed to SS1 and SS8 did not hatch during the test period and only 33.33% of embryos exposed to SS7 did hatch. The obtained results suggest that embryos exposed to sites with elevated levels of PAH experience reduced hatch rates and high mortality. Several studies generally found reduced hatch rates, suggesting that several pollutant-bound sediments may hinder zebrafish embryo hatching and lead to high mortality rates (Abdullah et al., 2022; Marara & Palamuleni, 2019; Pang et al., 2022).

Using the hazard classification system, sediment samples collected from sites SS3, SS7, WS6, and WS8 were categorized as class IV, indicating that the sediment samples were highly acute toxic, which was expected as those sites showed elevated levels of PAHs (Fig. 2). In the study done by Seopela et al. (2016), sites regarded as highly toxic had high levels of HMW PAHs compared to the LMW PAHs, which made them toxic even though they had lower levels of PAHs overall. Hence, the presence of high levels of HMW PAHs in the above-mentioned sites compared to the LMW PAHs contributed to the observed high level of toxicity in sediment samples from those sites (Sharifi et al., 2022). Moreover, the following sediment samples collected at sites SS1, SS8, WS1, WS5, and WS7 were regarded as class III, meaning they were acutely toxic, while those collected from sites SS2, SS4, SS6, WS2, WS3, and WS4 were classified as class II, meaning they were slightly acutely toxic. Lastly, the sediment samples from sites SS5, SS9, and WS9 were classified as class I, indicating no acute toxicity, which does not pose any threat to aquatic animals.

#### Malformation rate of zebrafish embryos exposed to river sediments

Endpoints such as lack of somite formation, non-detachment of a tail, spontaneous movements, non-development of eyes, developmental retardation, lack of heart function, lack of blood circulation, edema formation, lack of pigmentation, and spinal deformations were evaluated as a form of examining the malformations of zebrafish embryos throughout the test period. Malformations over 10% were regarded as significant as described in the test Guidelines 236 (TG 236) (OECD, 2013). Embryos exposed to the negative control did not show any malformations, but an overall malformation of 16.67% (edema formation at 96 hpf and spinal deformation at 72 hpf) was observed in the positive control. The total average percentage malformations of embryos exposed to sediment samples across all sites ranged from 0 to 87.50% (Fig. 6). The lowest and the greatest malformations were observed at sites SS1 (0%) and SS7(87.50%), respectively.

Embryos exposed to SS7 (87.50%) had a high malformation rate compared to the rest of the sediment samples, indicating the presence of a high level of pollution associated with that site (Bozinovic et al., 2021). Site SS1 does not pose any risk to the aquatic animals exposed to that site as no malformation was observed. The highest malformation rate observed for embryos exposed to SS7 was mainly due to the lack of somite formation, developmental retardation, lack of blood circulation, and lack of pigmentation. Site SS7 was previously regarded as class IV from mortality results, implying that the sediment samples from that site were highly acutely toxic to zebrafish embryos. Furthermore, SS7 has been regarded as the most polluted site with elevated levels of PAHs (Fig. 2), leading to the high observed malformations, raising a serious concern with regard to the developmental stages of the embryos and the aquatic animals exposed to those samples (Tartaglione et al., 2023). The conclusion was drawn because the widespread presence of PAHs in the water body and the environment has been linked to genotoxicity, developmental toxicity, and cardiac toxicity in exposed organisms such as zebrafish (McCormick et al., 2022). Lack of somite formation was experienced by embryos exposed to SS5 to SS8, WS1 to WS2, and WS4 to WS8. Non-detachment of the tail was not experienced by any embryo exposed to sediment samples throughout the test period. Spontaneous movements were only observed in embryos exposed to WS1. Developmental retardation was observed in embryos exposed to SS3 to SS4, SS7, WS1 to WS3, and WS6 to WS8. Hence, most of the embryos in the current study experienced developmental retardation compared to other endpoints.

Lack of heartbeat and blood circulation was only evident in embryos exposed to SS3 to SS4 and SS7. The observations correlate to the delayed hatch rate observed by embryos exposed to those sites. Pericardial edema was observed in embryos exposed to SS2 and SS4 as well as WS2, while a lack of pigmentation was observed at SS4, SS7 to SS9, and WS3.

#### The effect of sediment samples on the cardiology of zebrafish embryos

**Fig. 7:**
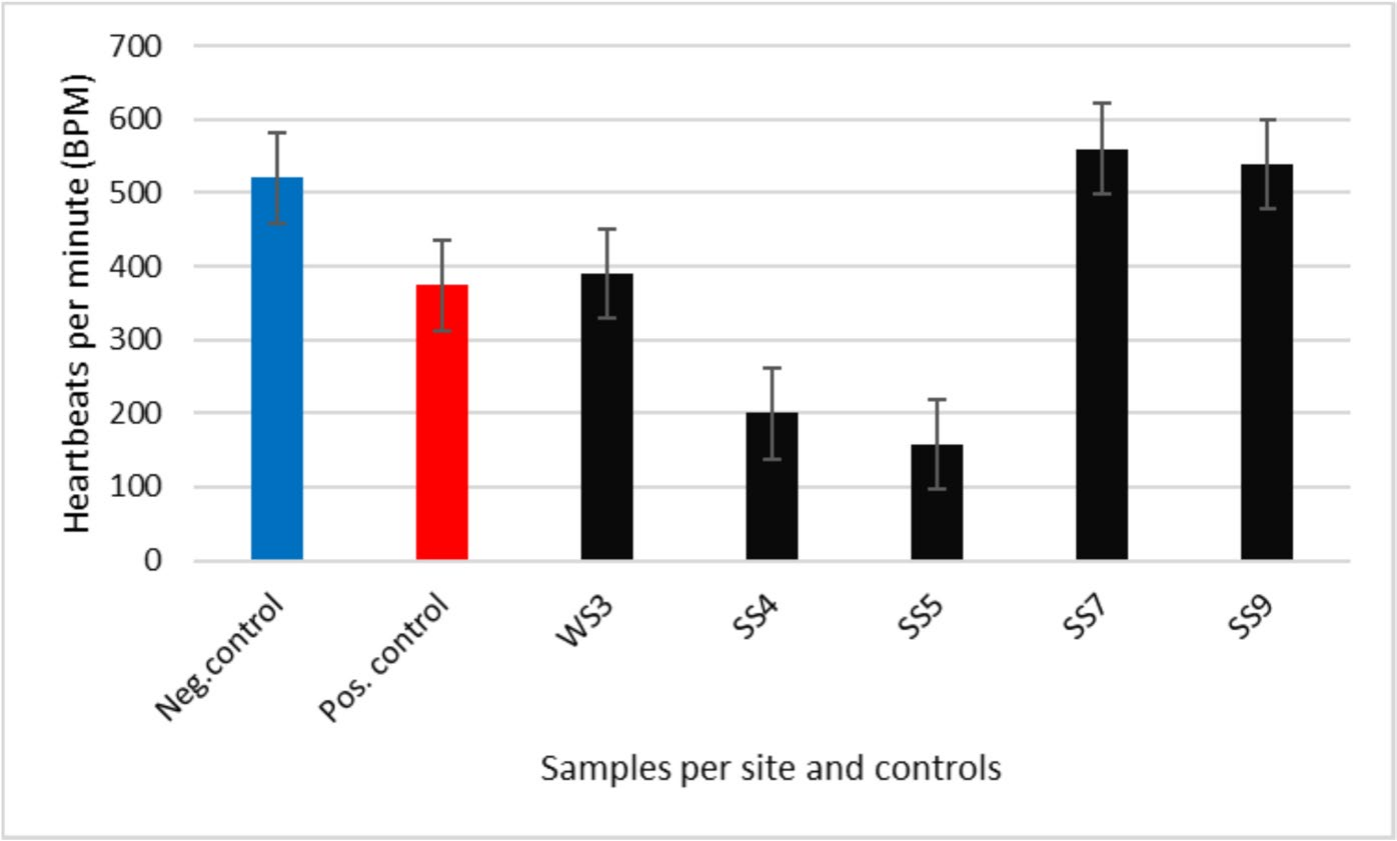
The heartbeat per minute of zebrafish embryos exposed to the sediment samples at 72 hpf, including the controls

**Fig. 8:**
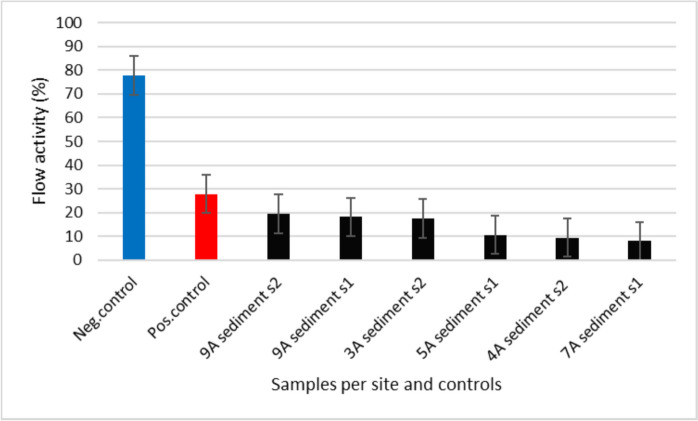
The blood flow activities of zebrafish embryos at 72 hpf exposed to sediment samples, including the controls

In addition to the evaluated malformations, the heartbeat (BPM) of the exposed zebrafish embryos was evaluated at 72 hpf. The BPM of zebrafish has been reported as one of the main functional endpoints in studies related to the toxicity of zebrafish embryos (cardiotoxicity) (Benslimane et al., 2020; McCormick et al., 2022; Ribeiro et al., 2020). The negative and positive controls were used as references for comparison with the sediment samples collected from Klip River. As expected, the BPM of embryos exposed to the negative control (520.45 BPM) was higher than the BPM of embryos exposed to the positive control (374.18 BPM), respectively (Fig. 8). Interestingly, the BPM of zebrafish embryos exposed to SS7 (560.01 BPM) and SS9 (539.03 BPM) were higher than those exposed to the negative control (Fig. 7). The observed phenomenon suggests a high metabolic rate in fish, possibly because of toxicant elimination, as heartbeat frequency is significantly influenced by the metabolic rate (Vincze et al., 2014). The BPM of embryos exposed to SS5 (158.18 BPM) and SS4 (200.00 BPM) were less than the BPM of the embryos exposed to negative control, indicating that the above-mentioned sites were polluted and negatively affected the heartbeats of zebrafish embryos, which pose a high risk to the survival of aquatic animals exposed to those sites. The study conducted by Cunha et al. (2020) demonstrated that zebrafish embryos exposed to BaP (HMW PAH) exhibited abnormalities in cardiac development. In addition, the study observed significant variations in the expression of genes associated with cardiovascular development, providing further evidence that disruption of gene expression plays an important role in the development of cardiac abnormalities brought on by PAH exposure. Hence, the observed reduced BPM was due to the toxicity of the above-mentioned sites, which also supports the observed lack of heart function in the malformation results associated with embryos exposed to SS4. However, SS5 was classified as class I, indicating no acute toxicity associated with that site, but the presence of HMW PAHs might cause the observed low BPM, which in turn negatively affected the cardiology of the zebrafish embryos. According to the study conducted by Seopela et al. (2016), individual combined effects of PAHs exposed to zebrafish embryos lead to genetic alterations and cardiac dysfunction. Hence, embryo exposure to PAH-contaminated sediments from the Klip River results in genetic alterations and reduced BPM (cardiac dysfunction), leading to the observed high mortality of zebrafish embryos and a reduced hatch rate.

#### The effect of sediment samples on the blood flow of zebrafish embryos

The blood flow activities of zebrafish embryos were also evaluated and monitored at 72 hpf. The validity of the test was confirmed by assessing the blood flow activities of zebrafish embryos exposed to the negative control (77.86%) and positive control (27.80%) (Fig. 8). High blood flow activities of embryos were observed in the negative control, compared to the positive control, suggesting that embryos in a medium without pollutants should have higher blood flow activities comparable to the negative control. All the test samples resulted in reduced blood flow, which ranged from 8.00 to 19.47%, with the lowest and highest blood flow experienced by embryos exposed to SS7 and WS2, respectively (Fig. 8).

Blood flow disturbances within the vessels of zebrafish embryos may be a sign of vascular abnormalities or heart muscle problems, which negatively affect the embryos and lead to high malformations resulting in elevated mortality (Benslimane et al., 2020; Cunha et al., 2020). In addition, according to the used hazard classification method, SS7 was classified as class IV (high acute toxicity), which corresponds to the reduced blood flow activities observed in embryos exposed to that site, implying that polluted sites consisting of elevated levels of PAHs negatively affect the blood flow activities of the zebrafish embryos (Cunha et al., 2020). The above findings suggested that zebrafish embryos exposed to a clean medium had higher blood circulation compared to those exposed to sediment samples, which supports the obtained malformation results categorization of lack of heartbeat and blood circulation experienced by embryos from SS7, leading to the observed reduced hatch rates and higher mortalities of embryos. Hence, most of the sediment samples tested impose a negative effect on the blood flow activities of the aquatic animals, which still need to be monitored by reducing the production of pollutants in the water and the environment.

## Conclusions

Sediment samples collected from the Klip River during the high and low flow seasons were found to contain the 16 US EPA priority PAHs, with the varying concentrations depending on the level of pollution per site. The findings of this study showed that sediment samples collected from Klip River during the low and high flow seasons had total concentrations of PAHs ranging from 2.35 to 7.41 mg/kg and 1.46 to 4.99 mg/kg, respectively. The low flow season sediment samples had higher concentrations of PAHs compared to the high flow sediment samples. Due to the various activities occurring during those seasons close to each site, there is a difference in the concentrations of PAHs that have been detected during high and low flow seasons. HMW PAH compounds were more prevalent than LMW PAHs in river sediments. Most PAHs detected in river sediments were above the maximum permissible level set by the Agency for Toxic Substances and Disease Registry (ATSDR), suggesting a possible concern for aquatic animals and human health. The sources of PAHs in the river sediments were identified using a diagnostic ratio. Several PAH ratios showed that the detected PAHs came from the combustion of petroleum, pyrogenic, and petrogenic sources occurring near the study area. These sources can be linked to the identified potential sources of pollution near each sampling site contaminating the river.

The toxicity study showed that areas with significant levels of PAHs had an adverse effect on embryo development, causing malformations. High levels of PAHs in river sediments result in high malformation rates, reduced hatch rates, and high mortality of zebrafish embryos. Embryos exposed to sediment samples contaminated with PAHs experienced low blood flow and heartbeat. Additionally, the risk that contaminated sediment samples pose to zebrafish embryos indicates that there might be risks experienced by biotic animals, livestock, agriculture, and people who use water for consumption.

According to the findings in the study areas under consideration, detected PAH levels are higher than the maximum permissible level. Thus, it is critical to continue implementing programs that aim to monitor and reduce the release of these PAHs in the environment and water systems.

## Supplementary Information

Below is the link to the electronic supplementary material.

Supplementary file1 (DOCX 202 KB)

## Data Availability

No datasets were generated or analyzed during the current study.
