# Supplementary material for: Seasonal variations, source apportionment, and risk assessment of polycyclic aromatic hydrocarbons (PAHs) in sediments from Klip River, Johannesburg, South Africa
Source: Environ Monit Assess. 2025 Feb 8;197(3):257. doi: 10.1007/s10661-025-13724-0 (PMC11807025; doi:10.1007/s10661-025-13724-0)
Supplement: Supplementary file 1 — Supplementary file1 (DOCX 202 KB) [file 10661_2025_13724_MOESM1_ESM.docx]

**Supplementary Information (SI)**

**Seasonal variations, source apportionment and risk assessment of polycyclic aromatic hydrocarbons (PAHs) in sediments from Klip River, Johannesburg, South Africa**

Samuel Makobe, Mathapelo P. Seopela, Abayneh A. Ambushe^*^

Department of Chemical Sciences, University of Johannesburg, P.O. Box 524, Auckland Park 2006, Johannesburg, South Africa

*Corresponding author: [aambushe@uj.ac.za](mailto:aambushe@uj.ac.za) (Abayneh A. Ambushe) Tel.: +27 11 559 2329; fax: +27 11 559 2819.

Samuel Makobe: Department of Chemical Sciences, University of Johannesburg, P.O. Box 524, Auckland Park 2006, Johannesburg, South Africa

Email: [samuelmakobe@gmail.com](mailto:samuelmakobe@gmail.com)

Mathapelo P. Seopela: Department of Chemical Sciences, University of Johannesburg, P.O. Box 524, Auckland Park 2006, Johannesburg, South Africa

Email: [mpseopela@uj.ac.za](mailto:mpseopela@uj.ac.za)

**Journal**: Environmental Monitoring and Assessment

- 1. *Reagents and consumables*

Acetone (≥ 99%), hexane (≥ 95%), and dichloromethane (≥ 99.5%) were all HPLC-grade and purchased from Sigma-Aldrich (Taufkirchen, Germany). Nitric acid (35%), copper powder, and anhydrous sodium sulphate (Na_2_SO_4_) were purchased from Sigma-Aldrich (Taufkirchen, Germany). An EPA 610-N PAH kit, comprising the 16 US EPA priority PAHs, namely; naphthalene, acenaphthylene, acenaphthene, anthracene, fluoranthene, phenanthrene, fluorene, pyrene, benzo(b)fluoranthene, benz(a)anthracene, chrysene, benzo(k)fluoranthene, benzo(a)pyrene, dibenzo(ah)anthracene, benzo(g,h,i)pyrene, and indeno(1,2,3-c,d)pyrene and internal standards naphthalene-D8, acenaphthene-D10, chrysene-D12, and perylene-D12 were bought from Supelco (Pennsylvania, USA). The standard solutions for calibration curves of 16 priority PAHs ranged from 0.50 to 1.00 mg/L. Calibration curves of 16 PAHs were constructed using the internal standard method at a final concentration of 1 mg/L. Lastly, the certified reference material of sediment (CRM-104) used for confirming the accuracy of the method was purchased from Sigma-Aldrich (Taufkirchen, Germany). Pure helium gas (99.99%) was used as a carrier gas (Afrox, Johannesburg, RSA).

- 1. *Apparatus and instrumentation*

Sediment samples were dried using a BK-FD10S freeze dryer (Biobase Biodustry, Jinan, China). A rotary evaporator (Buchi-evaporator R-200), equipped with a V-700 vacuum pump and a heating bath set at 40 °C, was used to concentrate the extracts and it was purchased from Labotec SA (Johannesburg, RSA). A Reacti-Vap™ Evaporating Unit used to control a gentle stream of nitrogen for the evaporation of the solvent in the extracts was purchased from Pierce (Illinois, USA). A Vortex-Genie 2 (G560E model) purchased from Lasec SA (Cape Town, RSA), was used to mix the vials containing samples. Extraction of PAHs in river sediments was conducted using a CEM MARS Microwave System (LabX, Midland, ON, Canada). An Agilent 7820A GC-FID (Santa Clara, USA) was used for identification and quantification of PAHs in river sediments. Separation of the PAHs was achieved using an Agilent J & W HP-5 column (Santa Clara, USA). A multi-linking ZebTec housing system (Tecniplast, Birmingham, Italy), was used for housing the male and female zebrafish. The mating process for zebrafish took place in an iSPAWN Zebrafish tank (Tecniplast, Birmingham, Italy). An incubator set at a temperature of 26 ± 1 °C (Sigma-Aldrich Chemie GmbH, Taufkirchen, Germany) was used to make sure that sediment samples on the plates were not tempered before the insertion of embryos.

**Table A1**

Column details and operating conditions for GC-FID

| Column | |
| --- | --- |
| Length of the column | 30 m |
| Thickness of the film | 0.25 µm |
| Internal diameter | 320 µm |
| Operating conditions | |
| He flow rate | 25 mL/min |
| Make up gas | Hydrogen |
| Purge flow to split vent | 30 mL/min at 0.5 min |
| H_2_ flow rate | 30 mL/min |
| Air | 400 mL/min |
| Average velocity | 38.369 cm/sec |
| FID temperature | 1. °C |

*2.7. Analytical figures of merit*

The calibration curves revealed a good linearity since the coefficient of determination (R^2^) ranged from 0.9989 to 0.9998, with Flu having the highest R^2^ value and BAnt having the lowest R^2^ value. The limit of detections (LODs) were between 0.0175 and 0.0454 mg/kg and the limit of quantifications (LOQs) ranged between 0.0582 and 0.151 mg/kg for the determination of PAHs in sediment samples, respectively (Table A2). The obtained LODs and LOQs were comparable to the one reported by other studies (Marara & Palamuleni, 2019; Mogashane et al., 2020).

**Table A2**

The retention times, R^2^, LODs, and LOQs for the PAH analysis by GC-FID

| PAHs | Retention  time (min) | R^2^ | LOD  (mg/kg) | LOQ  (mg/kg) |
| --- | --- | --- | --- | --- |
| Nap | 14.5 | 0.9996 | 0.0276 | 0.0920 |
| Acy | 19.0 | 0.9994 | 0.0334 | 0.111 |
| Ace | 19.5 | 0.9991 | 0.0420 | 0.140 |
| Flu | 20.8 | 0.9998 | 0.0175 | 0.0582 |
| Phe | 23.0 | 0.9994 | 0.0333 | 0.111 |
| Ant | 23.1 | 0.9996 | 0.0286 | 0.0955 |
| Fln | 26.5 | 0.9992 | 0.0383 | 0.128 |
| Pyr | 27.5 | 0.9996 | 0.0267 | 0.0889 |
| Bant | 34.2 | 0.9989 | 0.0454 | 0.151 |
| Chr | 34.4 | 0.9991 | 0.0412 | 0.137 |
| BbF | 39.3 | 0.9993 | 0.0365 | 0.122 |
| BkF | 39.4 | 0.9991 | 0.0418 | 0.139 |
| BaP | 40.6 | 0.9997 | 0.0253 | 0.0845 |
| InP | 45.2 | 0.9995 | 0.0301 | 0.100 |
| DahAnt | 45.5 | 0.9994 | 0.0339 | 0.113 |
| BghiP | 46.5 | 0.9990 | 0.0439 | 0.146 |

The percentage recoveries of the 16 PAHs extracted from CRM-104 ranged from 80.7 to 118%, with InP having the lowest percentage recovery and Fln having the highest percentage recovery, respectively (Table A3). The low percentage recoveries could be attributed to the LMW of the target compounds, which may have been lost during the evaporation process (Awe et al., 2020; Olayinka et al., 2018), where LMW PAHs (Nap-Ant) accounted for percentage recoveries of 82.4 to 103% and HMW PAHs (Fln-BghiP) accounted for 80.7 to 118% (Table 3). This is in agreement with acceptable percentage recoveries of over 80% compared to other studies using the same extraction method (Mekonnen et al., 2015; Mogashane et al., 2020; Seopela et al., 2016). The %RSD obtained for this study ranged from 0.672 to 1.94%, indicating good instrumental precision (RSD˂5.0%) (Alexandrino et al., 2024; Carvalho et al., 2019) (Table 3). Therefore, it can be concluded that MAE is an effective method for extracting PAHs from river sediments.

**Table A3**

Measured and certified concentrations, and percentage recoveries of PAHs extracted from CRM-104 by MAE and %RSD

| PAHs | Measured  values (µg/kg) | CRM-104 certified values (µg/kg) | %Recovery | %RSD |
| --- | --- | --- | --- | --- |
| Nap | 419 ± 4.4 | 418 ± 40 | 100 | 1.05 |
| Acy | 459 ± 3.8 | 557 ± 64 | 82.4 | 0.828 |
| Ace | 492 ± 7.1 | 478 ± 109 | 103 | 1.44 |
| Flu | 288 ± 5.5 | 340 ± 38 | 84.7 | 1.91 |
| Phe | 497 ± 4.8 | 510 ± 72 | 97.5 | 0.966 |
| Ant | 361 ± 4.3 | 393 ± 48 | 91.9 | 1.19 |
| Fln | 357 ± 2.4 | 303 ± 34 | 118 | 0.672 |
| Pyr | 335 ± 3.1 | 350 ± 47 | 95.7 | 0.925 |
| BAnt | 94.0 ± 1.2 | 110 ± 11 | 85.5 | 1.28 |
| Chr | 227 ± 1.9 | 231 ± 48 | 98.3 | 0.837 |
| BbF | 306 ± 2.4 | 318 ± 49 | 96.2 | 0.784 |
| BkF | 88.0 ± 1.7 | 95.1 ± 16.6 | 92.5 | 1.93 |
| BaP | 175 ± 3.4 | 159 ± 23 | 110 | 1.94 |
| InP | 96.0 ± 1.1 | 119 ± 29 | 80.7 | 1.15 |
| DahAnt | 212 ± 1.8 | 219 ± 17 | 96.8 | 0.849 |
| BghiP | 101 ± 1.9 | 103 ± 34 | 98.1 | 1.88 |

*2.8. Determination of PAHs in river sediments by GC-FID*

The chromatogram shown in Fig. A1 was obtained for the 16 US EPA priority PAHs, which inclde Nap, Acy, Ace, Flu, Phe, Ant, Fln, Pyr, BAnt, Chr, BbF, BkF, BaP, InP, DahAnt, and BghiP, as well as the four internal standards (Nap-D8, Ace-D10, Chr-D12 and Per-D12), from the analysis of a 1 mg/L standard solution, by GC-FID. With the exception of BAnt and Chr, the remaining target analytes and internal standards were well resolved. Each compound was identified by their retention times, confirmed by analysis of the individual standards (Table A2).


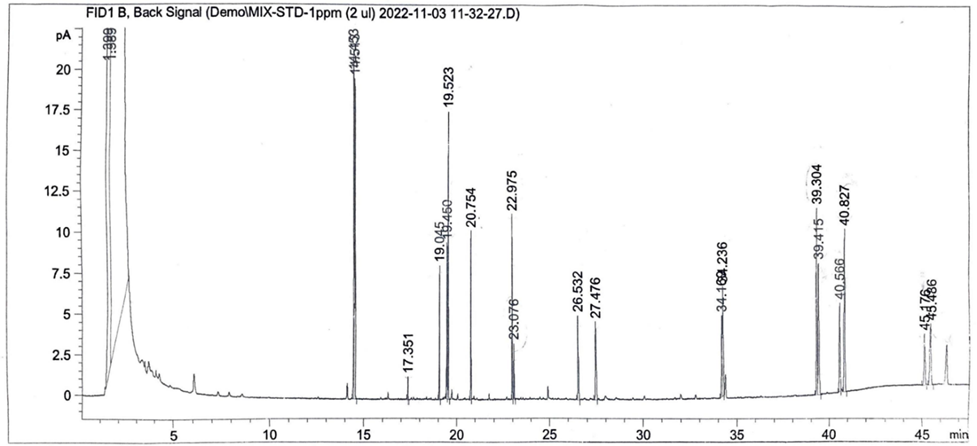


**Fig. A1**. Chromatogram of the detected 16 PAHs and 4 internal standards

**Table A4**

Hazard classification system for natural and wastewater (Ahad et al., 2020).

| Class | Percentage effect (PE) | Hazard |
| --- | --- | --- |
| Class I | ≤ 20% in all used biotests | No acute hazard |
| Class II | 20% ≤ PE ≤50% at least one biotest | Slight acute hazard |
| Class III | 50% ≤ PE ≤ 100% at least one biotest | Acute hazard |
| Class IV | PE = 100% in at least one biotest | High acute hazard |
| Class V | PE = 100% in all used biotests | Very high acute hazard |
